# Identification of bacterial communities associated with needle mushroom (*Flammulina filiformis*) and its production environment

**DOI:** 10.3389/fmicb.2024.1429213

**Published:** 2024-12-17

**Authors:** Zhenghui Liu, Yunlong Cong, Frederick Leo Sossah, Hongyan Sheng, Yu Li

**Affiliations:** ^1^Engineering and Research Center for Southwest Bio-pharmaceutical Resources of National Education Ministry, Guizhou University, Guiyang, China; ^2^Engineering Research Center of Edible and Medicinal Fungi, Ministry of Education, Jilin Agricultural University, Changchun, China; ^3^Institute of Edible Fungi, Guizhou University, Guiyang, China; ^4^Research Institute of Science and Technology, Guizhou University, Guiyang, China; ^5^Council for Scientific and Industrial Research (CSIR), Oil Palm Research Institute, Coconut Research Programme, Sekondi, Ghana; ^6^Department of Plant Pathology, Washington State University, Pullman, WA, United States

**Keywords:** *Flammulina filiformis*, cultivation environment, 16S amplicon, bacterial diversity, cultivable bacteria, pathogenicity determination

## Abstract

*Flammulina filiformis* is an important edible and medicinal mushroom widely cultivated in East Asia, with its quality and health strongly influenced by associated microbial communities. However, limited data exist on the bacterial communities associated with *F. filiformis* cultivation in Chinese farms. This study investigated bacterial communities associated with *F. filiformis* and its production environment using high-throughput 16S rRNA gene amplicon sequencing and culture-dependent methods. A total of 42 samples were collected from farms in Jilin and Guizhou provinces, China, for microbial community profiling. The analysis revealed diverse bacterial phyla, including *Proteobacteria*, *Firmicutes*, *Bacteroidetes*, *Actinobacteria*, and *Cyanobacteria*. Genera such as *Pseudomonas*, *Lactobacillus*, *Acinetobacter*, *Flavobacterium*, and *Phyllobacterium* were identified, with notable regional variations in the relative abundance of *Pseudomonas* and *Lactobacillus*. Pathogenic species, including *Pseudomonas tolaasii*, *Ewingella americana*, *Stenotrophomonas maltophilia*, *Pseudomonas* sp., *Lelliottia amnigena*, and *Janthinobacterium lividum*, were identified through phenotypic, biochemical, and molecular analyses. Pathogenicity tests confirmed the disease-causing potential of *P. tolaasii*, *E. americana*, and *J. lividum* in *F. filiformis*. These findings highlight regional differences in bacterial community composition and emphasize the need for tailored management practices. This study contributes to safe, high-quality mushroom cultivation and provides insights into improved cultivation practices, including Mushroom Good Agricultural Practices (MGAP).

## Introduction

*Flammulina filiformis*, classified as *Fungi*, *Basidiomycota*, *Agaricomycetes*, *Agaricales*, *Physalacriaceae*, *Flammulina* (Wang et al., 2018), is an important commercial mushroom valued for its high nutritional and medicinal properties (Yan et al., 2019a). The fruiting body of *F. filiformis* is rich in carbohydrate, protein, amino acids (Kalač, 2013). It also contains bioactive compounds that offer various health benefits, including anti-tumor, anti-atherosclerotic, antioxidant, and anti-aging effects (Rahman et al., 2015; Tang et al., 2016). *F. filiformis* accounts for over 10% of global mushrooms production, with China being the leading producer, contributing significantly to both domestic consumption and exports (Royse et al., 2017; Wang et al., 2018).

In recent years, the global demand for *F. filiformis* has increased due to its applications in traditional and functional foods, nutraceuticals, and as a source of bioactive compound in pharmaceuticals. Advances in production techniques, including innovations in substrate composition, cultivation technology, and quality control measures, have enhanced yield, disease resistance, and sustainability (Jayaraman et al., 2024; Sangeeta et al., 2024; Dong et al., 2022). The growing consumer preference for functional foods with health-promoting properties has reinforced *F. filiformis* as a vital component of the mushroom industry.

Microbial communities play a crucial role in the growth, development, and quality of mushrooms, including *F. filiformis* (Wang et al., 2018). These communities facilitate the breakdown of organic matter and nutrient cycling within the mushroom substrate, thereby providing essential nutrients for mushroom growth (Kalač, 2013). Microbial interactions also influence the flavor, aroma, and nutritional value of mushrooms through the production of enzymes and volatile compounds (Rahman et al., 2015; Tang et al., 2016).

However, certain microbial species can negatively impacts mushroom yield and quality, reducing crop productivity and affecting product shelf-life (Liu et al., 2022). Additionally, the cultivation environment can harbor microbes that pose food safety risks, such as *Listeria*, which may be harmful to humans (Li et al., 2021; Chen et al., 2014). Conversely, beneficial microbial communities can promote disease resistance by outcompeting harmful pathogens (Han et al., 2012; Wu et al., 2016). Studying these communities in both the mushroom, mushroom substrate and cultivation environment enables identification key microbial players that enhance growth, yield, and disease resistance, as well as potential sources of disease inoculum. Understanding and managing microbial communities is essential for optimizing yield, enhancing quality, and ensuring sustainable mushroom production (Berendsen et al., 2012).

To ensure safety and quality in mushroom cultivation, it is essential to adhere to Mushroom Good Agricultural Practices (MGAP), which include implementing Hazard Analysis and Critical Control Points (HACCP) protocols (Jin et al., 2008; Su et al., 2023). These practices encompass key aspects of cultivation, such as growing conditions, substrate preparation, hygiene, pest management, and harvesting. As an integral component of MGAP, HACCP identifies critical control points to mitigate potential hazards (Li and Xu, 2022). In China, the adoption of HACCP reflects a commitment to producing safe, high-quality mushrooms (Jin et al., 2008). The effectiveness of MGAP protocols, including HACCP, has been demonstrated in Chinese mushroom factories, ensuring consistent product quality (Jin et al., 2008; Li and Xu, 2022; Su et al., 2023).

Studying the structure and diversity of microbial communities is essential for mining microbial resources, understanding their functions, and elucidating the relationship between microbial communities and their habitats. Previous studies have highlighted the impact of casing composition and microbiota on disease development in *Agaricus bisporus* (Berendsen et al., 2012). The advent of metagenomics and advances in sequencing technology have made 16S rRNA gene sequencing a valuable method for analyzing microbial community diversity and composition (Youssef et al., 2009; Caporaso et al., 2011; Hess et al., 2011). The 16S rRNA gene is frequently used for bacterial phylogenetic analysis and classification, revealing species-specific characteristics.

Although 16S rRNA gene sequencing has been successfully applied to study microbial communities and dynamics in the cultivation of edible fungi, mushrooms and truffles (Benucci and Bonito, 2016; Vos et al., 2017; Carrasco et al., 2019), studies specifically examining the microbial communities associated with *F. filiformis* in its cultivation environment and factory settings are lacking. While some studies have examined the microbial communities associated with *F. filiformis* fruiting bodies (Wei et al., 2021), the microbial dynamics within the cultivation environment remain underexplored. Therefore, analyzing the microbial community structure and diversity related to *F. filiformis* cultivation is essential for the effective production management, ensuring food safety, preserving biodiversity, and maintaining ecosystem health.

This study adopted a comprehensive approach to investigate the microbial community structure and diversity within the cultivation and production environments of *F. filiformis* across two industrial-scale mushroom production facilities. Bacterial community composition was analyzed using high-throughput 16S rRNA gene amplicon sequencing. Additionally, traditional culture-dependent methods were employed to isolate and identify specific bacteria associated with diseases affecting *F. filiformis*. By integrating these techniques, a detailed understanding of the microbial dynamics within the cultivation and production environment of *F. filiformis* was obtained. This approach enabled the identification and characterization of bacterial species involved in disease development, contributing to deeper understanding of the factors affecting the health and productivity of *F. filiformis*.

## Materials and methods

### Study site and experimental design

The study was conducted in 2016 and 2017 at two industrial-scale *F. filiformis* mushroom production facilities in China. The first facility, Changchun Gaorong Biotechnology Co., Ltd. (44.20°N, 125.24°E), is located in Changchun, Jilin Province, while the second facility, Weining Xuerong Biotechnology Co., Ltd. (26.55°N, 104.14°E), is situated in Bijie City, Guizhou Province. These facilities were selected to represent distinct geographical regions and diverse climatic conditions. Both were dedicated indoor cultivation sites equipped with climate control systems, lighting, shelving or racks for mushroom trays, and automated conveyor system. While both facilities shared centralized monitoring and control systems for humidity, ventilation, and airflow, they differed in their design, layout, and production procedures.

A total of 42 samples were collected over 2 years, comprising seven groups at each location. Each group included three sub-samples used as biological replicates. Multiple samples from the same source group were combined to create a bulked sample, which was subsequently divided into three sub-groups to generate three biological replicates for that specific group. The groups from Changchun were labeled C1–C7, while those from Bijie were labeled G1–G7. Samples sources included: C1 and G1 (healthy *F. filiformis* fruiting bodies), C2 and G2 (diseased fruiting bodies of *F. filiformis*), C3 and G3 (cultivation substrate), C4 and G4 (humidified water reservoir), C5 and G5 (floor water from the mushroom facility), C6 and G6 (humidifier vent), and C7 and G7 (fresh air intake duct). All samples were transferred to sterilized 10 mL centrifuge tubes, rapidly frozen in liquid nitrogen, and stored at −80°C at the Engineering Research Center of the Ministry of Education, located at Jilin Agricultural University in Changchun, China.

### 16S rRNA amplicon metagenomic sequencing for bacterial community analysis

#### Library preparation and sequencing

DNA was extracted from all sample groups using the cetyltrimethylammonium bromide (CTAB) method (Clarke, 2009). DNA quantity and quality were assessed using a Nanodrop 2000 spectrophotometer (Thermo Electron Corporation, Waltham, MA, United States) and a 1.0% agarose gel, respectively. The fourth hypervariable (V4) region of the 16S rRNA gene was amplified using Phusion^®^vHigh-Fidelity PCR Master Mix (New England Biolabs, Ipswich, MA, United States) using primers 515F (5′-CCTACGGGAGGCAGCAG-3′) and 806R (5′-GGACTACHVGGGTATCTAAT-3′) each containing a 12-bp barcodes (Caporaso et al., 2011). PCR products were pooled based on their concentrations and separated on 1% agarose gels. The resulting PCR products were purified using GeneJET Gel Extraction Kit (ThermoFisher Scientific, Waltham, MA, United States). Library construction was performed using the Ion Plus Fragment Library Kit (ThermoFisher Scientific, Waltham, United States) according to the manufacturer’s instructions. Library quality was evaluated using Qubit 2.0 Fluorometer (ThermoFisher Scientific, Waltham, MA, United States) and an Agilent Bioanalyzer 2100 system (Agilent Technologies, Santa Clara, CA, United States). Finally, the 16S rRNA sequences for all 42 samples were generated using an Ion S5^™^ XL sequencer (ThermoFisher Scientific, Waltham, United States). DNA extraction, library preparation, and sequencing were performed by Novogene Biotech Company, located in Beijing, China.

### Sequence data analysis

Cutadapt software (V1.9.1)^1^ (Martin, 2011) was used to remove low-quality portions of the reads. Cleaned reads were then separated by sample based on the barcode sequences, with barcode and primer sequences trimmed off before preliminary quality control. The final clean reads were obtained by detecting and removing chimeric sequences (Haas et al., 2011) using VSEARCH and the species annotation database^2^ (Rognes et al., 2016).

Uparse software Uparse v7.0.1001^3^ (Edgar, 2013) was used to cluster cleaned reads at 97% similarity, generating Operational Taxonomic Units (OUTs) (He et al., 2015). The representative sequence with the highest frequency within each OTUs was selected. For species annotation of OTU sequences, the SILVA132 database^4^ (Quast et al., 2013), the small subunit (SSU) rRNA database (Kolisko et al., 2020), and the Mothur package (Schloss et al., 2009) were used, applying a threshold of 0.8–1 to obtain taxonomic information at various levels (kingdom, phylum, class, order, family, genus, species) (Wang et al., 2007). Data for each tested sample were standardized using the sample with the smallest data set as the reference, followed by analysis of Alpha diversity and Beta diversity. Qiime software (Version 1.9.1) was used to calculate the UniFrac distance and to construct the UPGMA (Unweighted Pair Group method with Arithmetic Mean) sample clustering tree. Non-metric multidimensional scaling (NMDS) analysis was performed using the vegan package in R, while differences between the Beta diversity index groups were analyzed with R software.

### Culture-dependent identification of pathogenic bacteria from diseased samples

Disease samples of *F. filiformis* (C2 and G2 groups), weighing 2 g each, were surface disinfection. The samples were first treated with 75% ethanol for 30 s, followed by immersion in 2% sodium hypochlorite solution for 1 min, then rinsed three times with sterile distilled water. The disinfected tissue blocks were mashed in sterile distilled water to prepare bacterial suspensions. These suspensions were streaked onto nutrient agar (NA) medium and incubated at 28°C in the dark for 48 h. Single colonies were selected, streaked NA at least three times to obtain pure cultures, which subsequently were stored in 70% glycerol at −80°C.

### Pathogenicity determination

To assess pathogenicity, single bacterial colonies were inoculated into 50 mL Erlenmeyer flasks containing 25 mL of Luria-Bertani (LB) liquid medium. The flasks were shaken at 28°C and 180 rpm for 12–18 h. Bacterial cells were collected by centrifugation, washed three times with sterile distilled water, and the suspension was adjusted to a concentration of 3 × 10^8^ CFU/mL.

White *F. filiformis* fruiting bodies, approximately 30 days old and at fruit body stage 1, were sourced from Changchun Gaorong Biotechnology Co., Ltd., a commercial mushroom production facility. Healthy fruiting bodies were inoculated with 20 μL of bacterial suspension at the contact point between the fruit bodies and the inner wall of the bottle mouth. Each strain was tested in triplicate bottles and sterile water was used as a control. All inoculated samples were maintained in a mushroom-growing room at Jilin Agricultural University, with a temperature 12–14°C, humidity of 95–98%, and controlled light conditions. Disease symptoms appeared on the fruiting bodies 12 h post-inoculation, followed by re-isolation of the pathogenic bacteria. The control samples treated with sterile distilled water remained disease-free. The pathogenicity test was repeated three times for result calidation.

### Morphological identification of pathogenic bacteria

The isolated pathogenic bacteria were cultured on NA and King’s B (KB) medium at 28°C in the dark for 48–72 h. Observations were made on the size, color, shape, and fluorescence of the bacterial colonies on each medium. Bacterial cells grown on NA medium for 24 h were further examined using transmission electron microscope and scanning electron microscope to assess cell shape, size, presence of flagella, and growth patterns.

### Biochemical identification of pathogenic bacteria

Bacteria cultures were grown overnight in 5 mL of liquid LB medium at 30°C, shake at 180 rpm for 12 h. The cultures were centrifuged at 25°C and 12,000 rpm for 1 min, and the supernatant was discarded before washing the bacterial pellet twice with sterile distilled water. Biochemical tests were performed on all isolated strains using API 20E and API 50CHE kits (BioMérieux, Marcy-l’Etoile, France) according to the protocols described by Mergaert et al. (1993). All tests were repeated three times to ensure reliability.

### 16S rRNA identification of pathogenic bacteria

Bacterial strains isolated from the C2 and G2 groups were cultured on NA medium for 24 h, after which single colonies were transferred to 50 mL conical flask containing 20 mL of LB liquid medium. The cultures were incubated at 28°C with shaking at 180 rpm for 12–18 h. Genomic DNA was extracted from liquid cultures using the BioFlux Biospin bacterial genomic DNA extraction kit (Bioflux, Beijing, China). The concentration and quality of the extracted DNA were assessed using a Shimadzu BioSpec-nano UV–Vis spectrophotometer (Shimadzu, Kyoto, Japan) and 1% gel.

The 16S rRNA gene was amplified using the primers 27F (5′-AGAGTTTGATCCTGGCTCAG-3′) and 1492R (5′-TACGGTTACCTTGTTACGACTT-3′) (Lane, 1991). The 25 μL PCR reaction mixture consisted of: 5 μL of 5 × TranStart Faspfu Bufferl, 2 μL of dNTPs (2.5 mM), 1 μL of forward primer, 1 μL of reverse primer, 1 μL of genomic DNA, 0.5 μL of TranStart Faspfu DNA Polymerase (5 U/μl), and 14.5 μL of ddH_2_O. The PCR protocol involved an initial denaturation at 94°C for 5 min; followed by 30 cycles of denaturation at 94°C for 1 min, annealing at 55°C for 30 s, and extension at 72°C for 1 min; with a final extension at 72°C for 5 min (Liu et al., 2020). PCR products were visualized using 1% agarose gel electrophoresis.

The PCR products were then purified and sequenced Sangon Bioengineering Co., Ltd. (Shanghai, China). The resulting 16S rRNA gene sequences were edited using BioEdit7.2 (Hall, 1999) and compared to sequences in the GenBank database using the BLAST tool.^5^ Sequences showing 100% similarity index were downloaded and aligned with ClustalW (Thompson et al., 1994) for multiple sequence alignment. A phylogenetic tree was constructed based on the 16S rRNA sequences of bacteria was constructed using MEGA11 (Tamura et al., 2021) with the Neighbor-Joining method and 1,000 bootstrap replications (Tamura et al., 2004).

### Statistical analysis

All statistical analyses were conducted using RStudio (version 4.2.1) and QIIME2. For microbial community data, OTU tables were transformed as needed, and relative abundances at the phylum, genus, and species levels were calculated. Alpha diversity indices (Shannon, Simpson, Chao1, and Observed OTUs) were computed using the “vegan” package (Oksanen et al., 2020). One-way ANOVA and the nonparametric Kruskal-Wallis test were used to assess differences in alpha diversity, with post-hoc comparisons conducted using Tukey’s HSD or Wilcoxon rank-sum tests.

Beta diversity was evaluated using PCoA based on Bray–Curtis dissimilarity to explore microbial community structure differences between sample groups. PERMANOVA and ANOSIM, conducted with the “adonis” function in “vegan,” tested for significant differences in community composition. NMDS was used to visualize these differences.

Pairwise PERMANOVA and ANOSIM were employed to detect significant differences across sampling sites and between healthy and diseased samples. Spearman correlation tests were applied to assess relationships between environmental variables and microbial diversity/composition. *p*-values were adjusted for multiple comparisons using the Benjamini-Hochberg correction. Key taxa contributing to differences in microbial diversity were identified using heatmaps generated with the “pheatmap” package.

## Results

### Characterization of diseased symptoms

**Figure 1:**
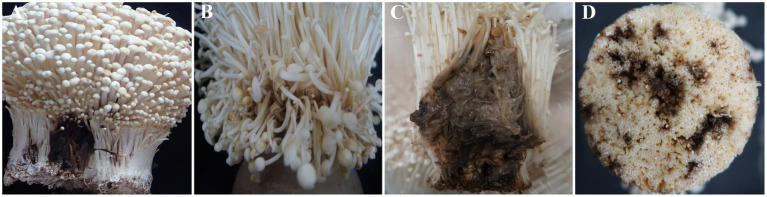
Four types of disease symptoms observed in *Flammulina filiformis* fruiting bodies. **(A)** Black root rot disease observed only in the C2 group. **(B)** Light brown rot disease observed in both C2 and G2 groups. **(C)** Dark brown rot disease observed only in the G2 group. **(D)** Black brown rot disease observed only in the G2 group. The C2 group was collected from Changchun, while the G2 group was collected from Bijie.

Symptoms of rot were observed on the mushroom fruiting bodies with four distinct types identified The C2 samples exhibited black rot and light brown rot symptoms (Figures 1A,B), while the G2 samples displayed light brown rot, dark brown rot, and black brown rot in their fruiting bodies (Figures 1B–D).

### 16S rRNA amplicon metagenomic sequencing for bacterial community analysis

#### Sequence analysis

**Figure 2:**
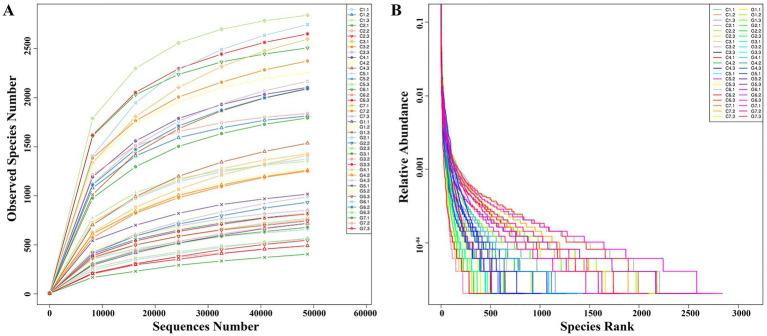
Rarefaction and rank abundance analysis for *Flammulina filiformis* and its cultivation environment. **(A)** Rarefaction curve showing the relationship between sequencing depth and the number of observed species. **(B)** Rank abundance curve displaying the distribution of species abundances within the microbial community.

Single-end sequencing was conducted on 42 samples using the Ion S5TM XL platform. After shear filtering and quality control, an average of 79,718 raw reads per sample were obtained, with 76,051 clean reads were retained post-chimera removal. The average read length was 252 bp, corresponding to the V4 region of the 16S rRNA gene. The mean GC content was 52.3%, with quality control efficiency of 95.5% (Supplementary Table S1). Rarefaction analysis confirmed sufficient sequencing depth, approaching saturation as the average read per sample approached 48,826, nearing saturation (Figure 2A). The observed increase in bacterial OTUs with higher sequencing depth demonstrated comprehensive coverage of the bacterial diversity. Rank abundance analysis showed a clear species rank pattern, where relative abundance decreased progressively, evident from the step-like structure in the rank abundance curve at lower abundance levels (Figure 2B).

### Operational taxonomic unit clustering analysis

**Figure 3:**
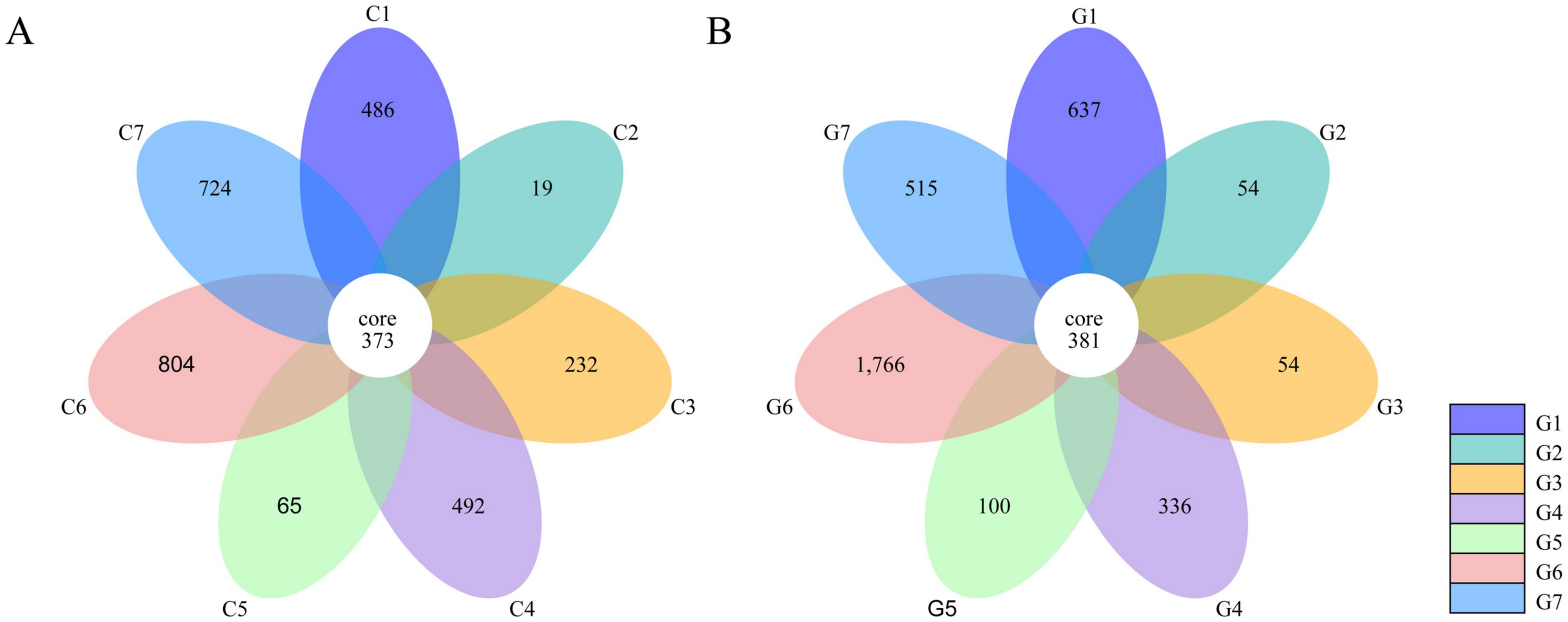
Common and unique operational taxonomic units (OTUs) based on 16S rRNA sequences in different groups of samples collected from *Flammulina filiformis* factories in Changchun **(A)** and Bijie **(B)**.

OTU clustering at a 97% identity threshold yielded 7,038 OTUs across all samples. The Changchun factory samples contained 3,195 OTUs, while the Guizhou factory samples had 3,843 OTUs. In the Changchun samples, 373 OTUs (11.7%) were shared among all groups (Figure 3A). Group C6 had the highest number of unique OTUs at 804 (25.2%), while group C2 had the fewest at 19 (0.6%). The remaining groups (C7, C4, C1, C3, and C5) had 724 (22.7%), 492 (15.4%), 486 (15.2%), 232 (7.3%), and 65 (2.0%) unique OTUs, respectively. In the Guizhou samples, 381 core OTUs (9.9%) were shared across all groups (Figure 3B). Group G6 had the highest number of unique OTUs at 1,766 (46.0%), while groups G2 and G3 had the lowest, with 54 (1.4%) each. Groups G1, G7, G4, and G5 had 637 (16.6%), 515 (13.4%), 336 (8.7%), and 100 (2.6%) unique OTUs, respectively.

### Bacterial community abundance

**Figure 4:**
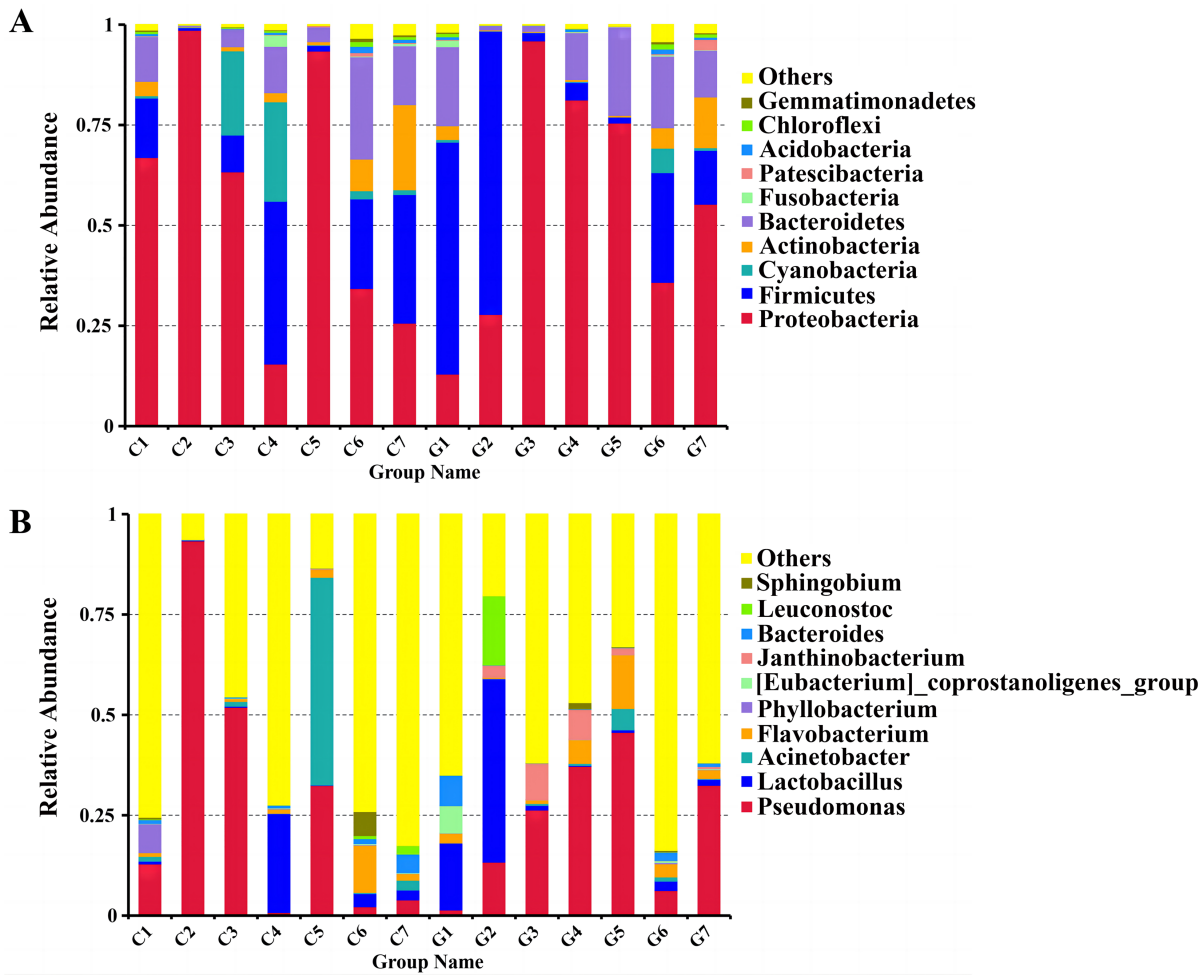
Comparison of bacterial communities at the phylum and genus levels. **(A)** Distribution of bacterial phyla. **(B)** Distribution of bacterial genera. Groups C1–C7 represent samples from Changchun, while groups G1–G7 represent samples collected from Bijie, as detailed in Supplementary Table S5. Each color in the figures corresponds to a specific phylum or genus, with relative abundance represented as a percentage.

Sequences were classified at various taxonomic levels, including phyla, classes, orders, families, and genera. The two most abundant phyla were *Proteobacteria* and *Firmicutes*, accounting for 78.27 and 17.01% of the sequences, respectively. *Proteobacteria* predominated in most samples, except for C4, C7, G1, and G2, where *Firmicutes* were more prevalent. The top 10 phyla identified were *Proteobacteria*, *Firmicutes*, *Bacteroidetes*, *Actinobacteria*, *Cyanobacteria*, *Acidobacteria*, *Chloroflexi*, *Fusobacteria*, *Patescibacteria*, and *Gemmatimonadetes* (Figure 4A; Supplementary Table S2). At the genus level, the 10 most abundant genera included *Pseudomonas*, *Lactobacillus*, *Acinetobacter*, *Flavobacterium*, *Phyllobacterium*, [*Eubacterium*]_coprostanoligenes_group, *Janthinobacterium*, *Bacteroides*, *Leuconostoc*, and *Sphingobium* (Figure 4B; Supplementary Table S3). In the Changchun factory samples, *Pseudomonas* was most abundant in C2, whereas *Acinetobacter* predominated in C5. In the Guizhou factory, *Lactobacillus* was the most prevalent in G2, while *Pseudomona*s was dominant in G5, G4, G7, and G3. A evolutionary tree analysis showed that *Pseudomonas* and *Lactobacillus* consistently exhibited high abundance across all samples (Supplementary Figure S1).

### Bacterial community diversity

#### Alpha diversity analysis

Alpha diversity was analyzed to assess microbial diversity and richness within in the samples. Various alpha diversity indices, including observed species, Shannon, Simpson, Chao1, ACE, goods coverage, and PD whole tree indices, were calculated for each sample group (Table 1). The observed species count ranged from 481 (C2) to 2,559 (G6), indicating differences in species evenness. The Shannon index values varied from 4.618 (C2) to 8.469 (C7). Simpson index values ranged from 0.835 (C4) to 0.987 (C7), reflecting variations in dominance. The Chao1 index ranged from 676.031 (C2) to 2685.340 (C7), while the ACE index varied from 753.418 (C2) to 2805.019 (C7). Goods coverage values ranged from 0.989 (C7) to 0.996 (C2), suggesting high sampling coverage. Finally, the PD whole tree index ranged from 53.775 (C2) to 221.501 (G6), indicating phylogenetic diversity differences between samples.

**Table 1:** Diversity and abundance indices of samples*.

Group	Observed_species	Shannon	Simpson	Chao1	ACE	Goods_coverage	PD_whole_tree
C1	1,513	6.370	0.939	1610.772	1637.890	0.995	141.585
C2	481	4.509	0.870	676.031	753.418	0.996	53.775
C3	1,173	5.490	0.926	1451.696	1501.058	0.993	111.498
C4	1,639	5.695	0.835	1932.930	1943.854	0.992	152.268
C5	751	5.329	0.930	992.135	1007.987	0.995	66.774
C6	1,971	8.188	0.980	2244.169	2218.387	0.993	163.961
C7	2,379	8.469	0.987	2685.340	2805.019	0.989	176.772
G1	1,678	6.787	0.949	1983.008	2001.021	0.992	152.913
G2	687	4.618	0.860	904.161	986.137	0.995	65.517
G3	739	5.059	0.907	947.845	1000.134	0.995	68.935
G4	1,226	6.208	0.935	1505.301	1576.240	0.993	112.444
G5	748	6.303	0.961	1095.703	1118.040	0.995	79.782
G6	2,559	8.440	0.983	2849.887	2844.811	0.991	221.501
G7	1,375	6.481	0.911	1572.678	1567.955	0.995	128.294

*The table presents a summary of diversity and abundance indices calculated for the samples. The indices included are as follows: observed species (obser.), Chao1 index, Shannon index, Simpson index, ACE (abundance-based coverage estimator), goods coverage, and PD_whole_tree index (phylogenetic diversity based on the whole tree).

### Beta diversity analysis

#### NMDS analysis

**Figure 5:**
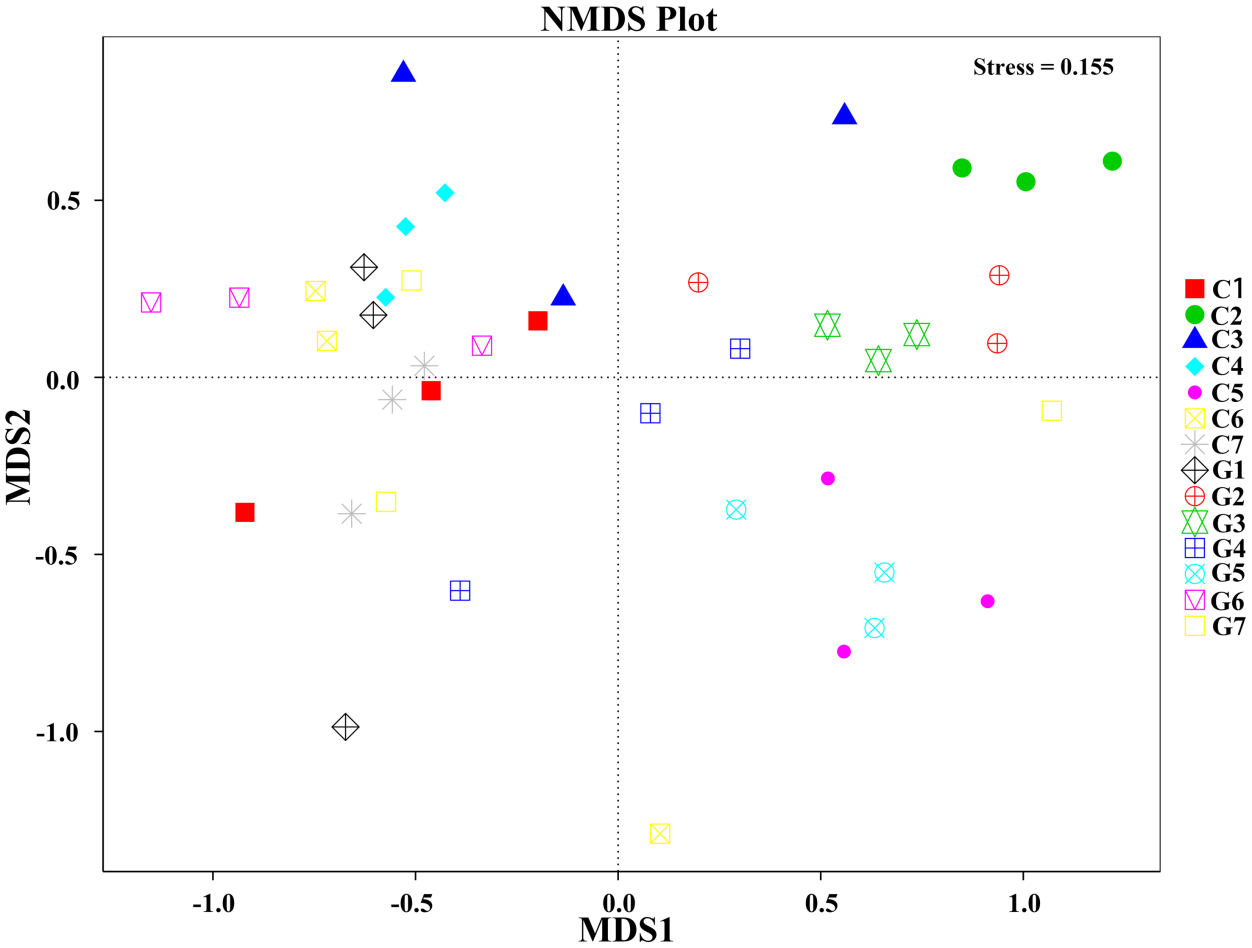
Non-Metric Multidimensional Scaling (NMDS) plot illustrating the relationship between samples based on operational taxonomic unit (OTU) data. Groups C1–C7 represent samples collected from Changchun, while groups G1–G7 correspond to samples collected from Bijie, as detailed in Supplementary Table S5. Samples within the same group are represented by the same shape and color. The distances between points in the plot indicate the degree of similarity or dissimilarity between the samples.

Non-metric Multidimensional Scaling (NMDS) analysis was conducted to explore the distribution patterns of samples in a two-dimensional space defined by NMDS1 and NMDS2 (Figure 5). Samples were labeled according to their respective group and sub-sample numbers. In the NMDS plot, group C (C1–C7) displayed a relatively compact distribution with some overlap among adjacent samples, whereas group G (G1–G7) exhibited a more dispersed distribution, reflecting greater variations. Distinct clustering patterns were observed for both group C and group G, although sub-samples within each group (including G1.1, G1.2, and G1.3) tended to cluster closely together.

### UPGMA clustering tree

**Figure 6:**
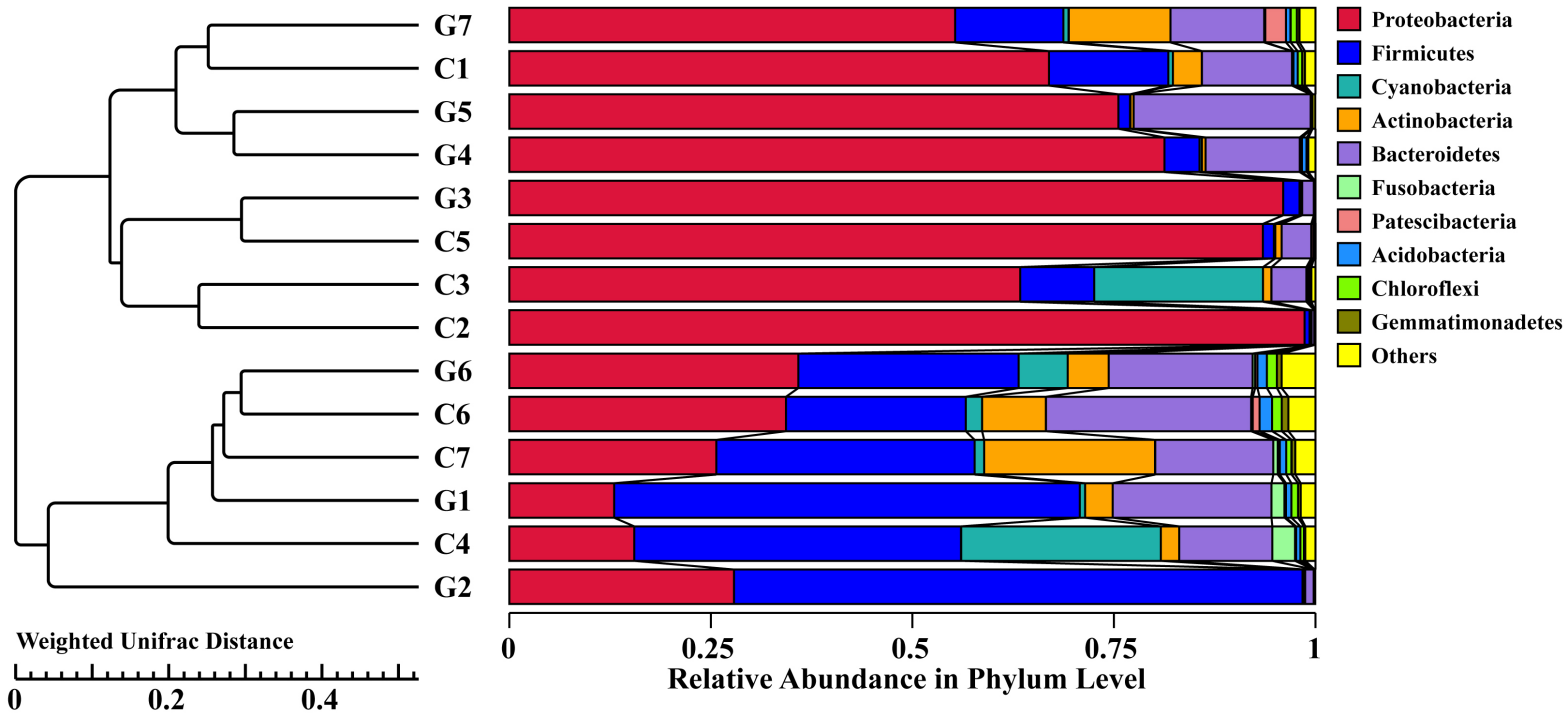
Hierarchical clustering tree diagram of samples based on operational taxonomic units (OTUs), constructed using the UPGMA clustering method and Weighted Unifrac distance. Groups C1–C7 represent samples collected from Changchun, while groups G1–G7 correspond to samples collected from Bijie, as detailed in Supplementary Table S5. Each color in the diagram corresponds to a specific phylum, with the relative abundance of each phylum represented as a percentage.

The UPGMA analysis, based on the weighted Unifrac distance metric, was used to evaluate phylogenetic relationships among the samples. The resulting tree demonstrated a complex clustering pattern, where samples from groups C (C1–C7) and G (G1–G7) formed mixed clusters rather than strictly separate ones (Figure 6). Notably, sub-samples G7, C1, and C4 formed a distinct sub-cluster, suggesting that the clustering pattern did not align strictly with the predefined group classifications.

### Isolation of bacteria and confirmation of pathogenicity

**Figure 7:**
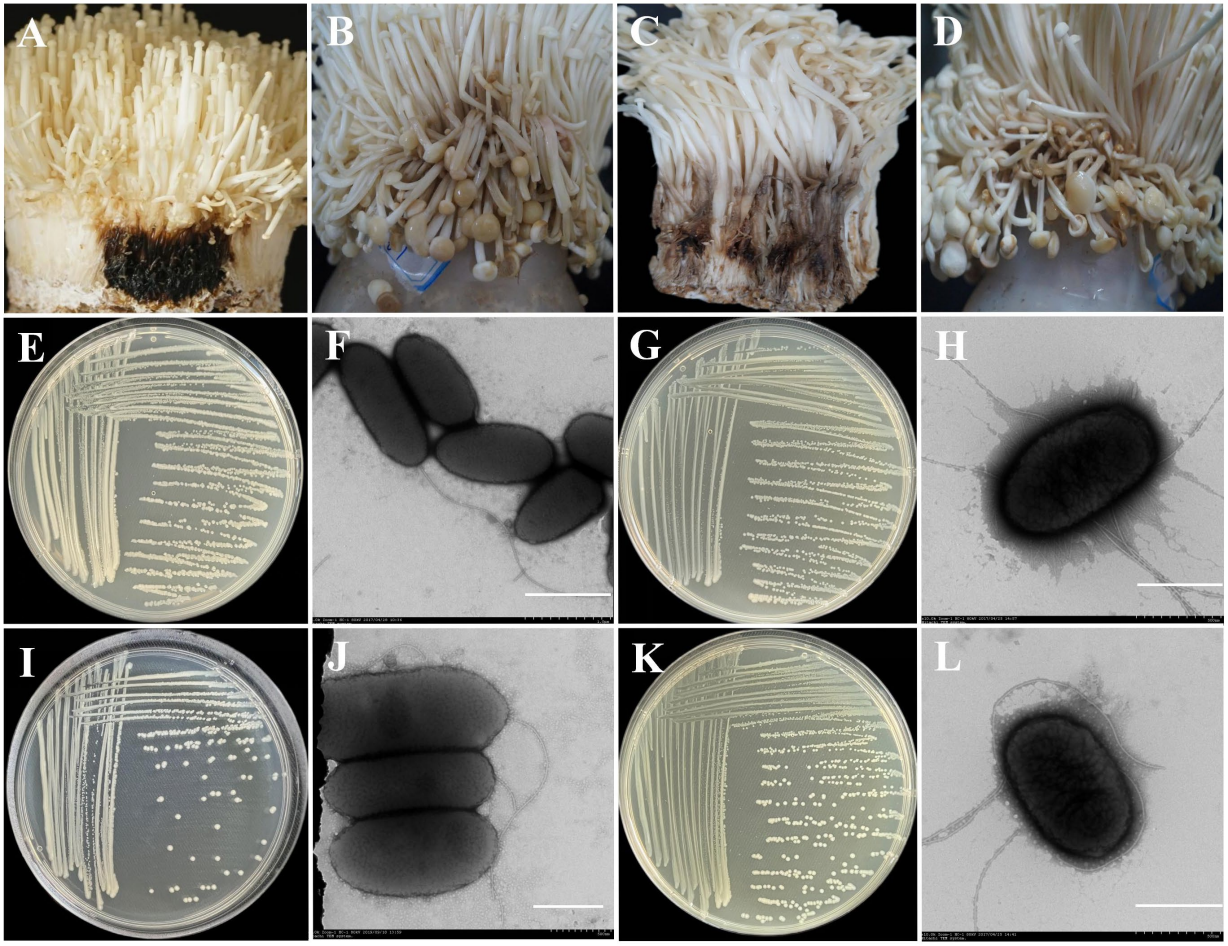
Pathogenicity and morphological characteristics of different pathogenic bacteria. **(A)** Inoculation with Pt11 bacteria suspension; **(B)** Inoculation with B2-1 bacteria suspension; **(C)** Inoculation with YC2-2 bacteria suspension; **(D)** Inoculation with GZ2-2 bacteria suspension; **(E)** Morphological characteristics of PT13 colonies cultured on NA plates at 28°C for 2 days; **(F)** Morphological characteristics of PT13 under a transmission electron microscope, (scale bar = 1 μm); **(G)** Morphological characteristics of B2-1 colonies cultured on NA plates at 28°C for 2 days; **(H)** Morphological characteristics of PT13 under a transmission electron microscope, (scale bar = 500 nm); **(I)** Morphological characteristics of YC2-2 colonies cultured on NA plates at 28°C for 2 days; **(J)** Morphological characteristics of YC2-2 under a transmission electron microscope, (scale bar = 500 nm); **(K)** Morphological characteristics of GZ2-2 colonies cultured on NA plates at 28°C for 2 days; **(L)** Morphological characteristics of GZ2-2 under a transmission electron microscope, (scale bar = 500 nm).

To evaluate the pathogenicity of the isolated bacteria, seven single colonies were obtained from two groups, C2 and G2. From group C2, B2-1, and PT13 were isolated, while group G2 yielded GZ1-3, GZ2-3, GZ2-2, GZ2-4, and YC2-2. Pathogenicity tests showed that PT13 and B2-1 from the C2 sample were pathogenic toward *F. filiformis*, whereas the control group inoculated with sterile water exhibited no disease symptoms. PT13 inoculation led to black rot within 24 h, accompanied by a darkening color and a strong putrid odor, replicating natural disease symptoms (Figure 7A). Similarly, B2-1 induced water-soaked lesions that changed from white to light brown within 2 days, progressively deepening in color over 4 days. The infected stalk became soft and unable to remain upright, consistent with typical disease manifestations (Figure 7B).

Of the six strains isolated from the G2 group, three demonstrated pathogenicity toward *F. filiformis*, while the others did not. Inoculation with YC2-2 resulted in rot appearing 2 days post-inoculation, transitioning from white to black-brown with a persistent putrid odor (Figure 7C). The symptoms produced by GZ2-2 were similar to those caused by B2-1 (Figure 7D).

To confirm the identity of the re-isolated pathogens from inoculated fruit bodies, morphological, physiological, biochemical, and 16S rRNA identification tests were conducted. These confirmed the characteristics of the inoculated bacteria, fulfilling Koch’s postulates and verifying their pathogenicity.

### Morphological identification of pathogenic bacteria

The PT13 colony on NA medium appeared round, milky white, convex, and smooth with regular edges (Figure 7E). The cells were rod-shaped, measuring 0.8–0.9 × 1.5–2.3 μm, highly virulent, and possess 1–2 flagella (Figure 7F). The B2-1 colony was white or milky white, round, sticky, convex, and smooth (Figure 7G). Its cells were short rods, measuring 0.8–1.0 × 1.5–1.8 μm, and had peripherally located flagella (Figure 7H).

The YC2-2 colony was white, round, with neat edges and convex surface (Figure 7I). Its cells were short rods, measuring 0.82–1.00 × 1.80–2.75 μm, with a single flagellum (Figure 7J). The GZ2-2 colony was white or milky white, round, sticky, convex, smooth, and had neat edges (Figure 7K). Its cells were short rods, measuring 1.5–1.8 μm × 0.8–1.0 μm, with peripherally located flagella (Figure 7L).

### Physiological and biochemical identification of pathogenic bacteria

The four strains of pathogenic bacteria examined in this study were identified as Gram-negative and exhibited a positive reaction for tobacco hypersensitivity. Each strain displayed distinct physiological and biochemical characteristics: PT13 tested positive for the white line reaction, arginine hydrolysis, and gelatin liquefaction but was negative for oxidase production, hydrogen sulfide (H_2_S) production, and indole production. PT13 metabolized carbon sources such as glucose, sodium citrate, D-ribose, rhamnose, arabinose, sorbitol, mannitol, arabinol, and pyruvate. However, it could not utilize urea, sucrose, esculin, gluconate, beta-galactoside, inositol, or maltose (Supplementary Table S4).

B2-1 exhibited positive results for contact enzyme production, tryptophan decarboxylase activity, nitrate reduction, and salicin reaction but was negative for gelatin hydrolysis, oxidase production, and indole reaction. It metabolized mannose, fructose, lactose, galactose, escin, acetate, and arabinol as sole carbon sources but could not utilize raffinose, glucose, mannitol, inositol, maltose, sucrose, or sorbose (Supplementary Table S4). GZ2-2 displayed similar characteristics with B2-1 (Supplementary Table S4). YC2-2 tested positive for catalase production, gelatin liquefaction, and arginine hydrolysis but was negative for the fluorescent reaction, oxidase reaction, indole reaction, H_2_S production, starch hydrolysis, nitrate reduction, lysine decarboxylase activity, ornithine decarboxylase activity, and tryptophan decarboxylase activity. YC2-2 utilized pyruvate, sodium citrate, esculin hydrate, urea, amygdalin, D-ribose, maltose, arabitol, and arabinose as sole carbon sources but could metabolize utilize rhamnose, sucrose, glucose, sorbitol, melibiose, or mannitol (Supplementary Table S4).

### 16S rRNA identification

The amplified 16S rRNA gene products from all eight purified strains ranged from 1,403 to 1,465 bp. Alignment with sequences in the GenBank database revealed high similarity, with seven strains showing 99–100% homology with *Pseudomonas tolaasii* (MH235995), *Ewingella americana* (CP048243), *Stenotrophomonas maltophilia* (KF941215), *Pseudomonas* sp. (KY623380), *Lelliottia amnigena* (MT634455), and *Janthinobacterium lividum* (KT767666). These findings elucidated the taxonomic classification of the analyzed strains.

**Figure 8:**
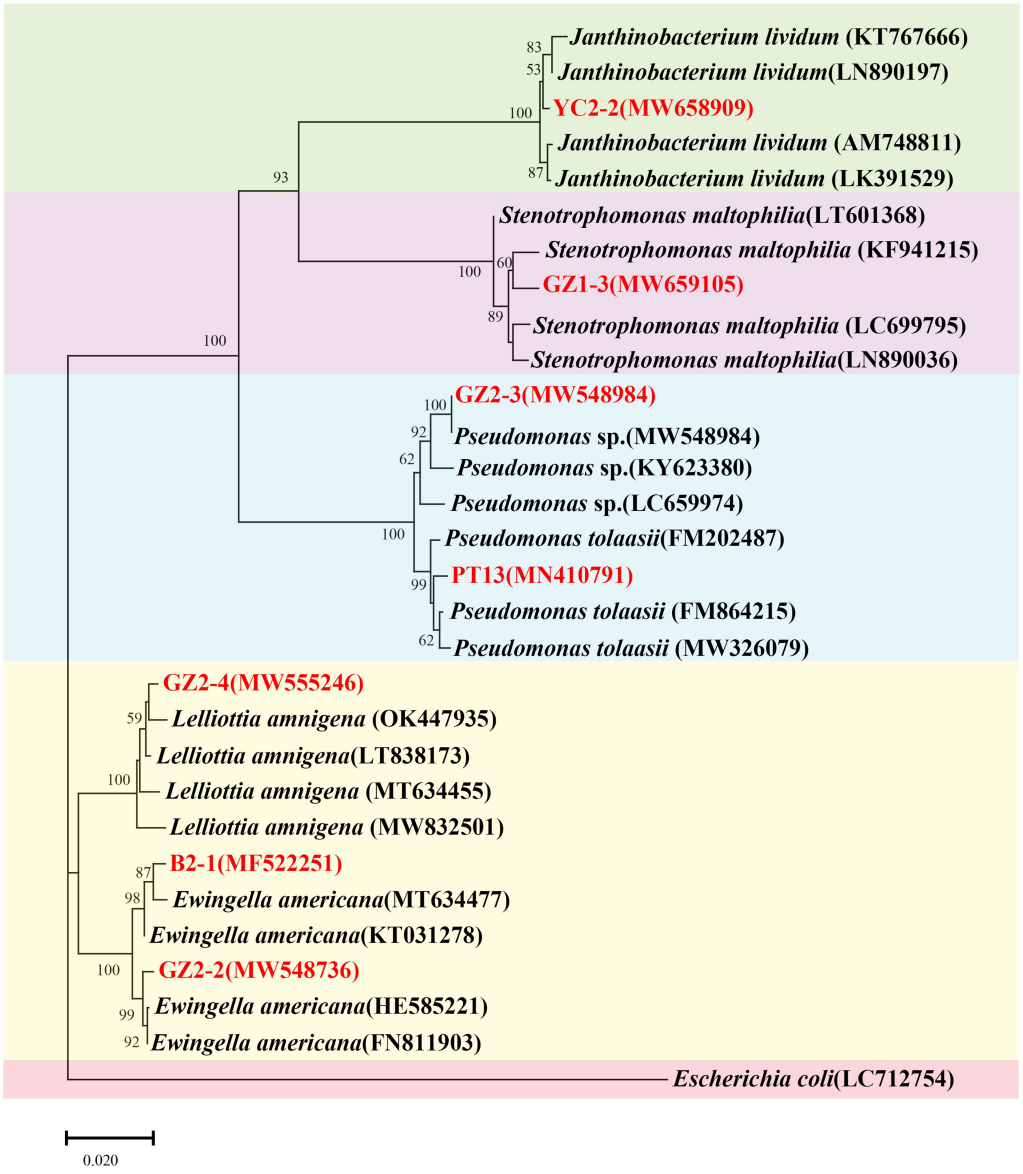
Neighbor-joining consensus phylogenetic tree based on partial 16S rRNA gene sequences of bacterial isolates obtained from factory cultivation environment and diseased fruiting bodies of *Flammulina filiformis*. Bootstrap values are based on 1,000 replicates. Strains analyzed in this study are highlighted in red. *Escherichia coli* (LC712754) was used as the outgroup.

Phylogenetic analysis confirmed the identity of strains YC2-2, GZ1-3, GZ2-3, PT13, GZ2-4, B2-1, and GZ2-2 as closely related to *J. lividum*, *S. maltophilia*, *Pseudomonas* sp., *P. tolaasii*, *L*. *amnigena*, and *E. americana*, respectively (Figure 8). These findings elucidated the taxonomic classification of the analyzed strains. The sequences of the seven strains were submitted to GenBank, and accession numbers were obtained (Table 2).

**Table 2:** Identification the strains isolated and purified from C2 and G2 by 16S rRNA amplification.

Strain number	Family	Species	City	Pathogenic assay	NCBI accession number
B2-1	*Enterobacteriaceae*	*Ewingella americana*	Changchun	+	MF522251
PT13	*Pseudomonadaceae*	*Pseudomonas tolaasii*	Changchun	+	MN410791
GZ1-3	*Xanthomonadaceae*	*Stenotrophomonas maltophilia*	Bijie	−	MW659105
GZ2-3	*Pseudomonadaceae*	*Pseudomonas* sp.	Bijie	−	MW548984
GZ2-2	*Enterobacteriaceae*	*Ewingella americana*	Bijie	+	MW548736
GZ2-4	*Enterobacteriaceae*	*Lelliottia amnigena*	Bijie	−	MW555246
YC2-2	*Oxalobacteraceae*	*Janthinobacterium lividum*	Bijie	+	MW658909

## Discussion

Microbial communities in the *F. filiformis* cultivation environment play a crucial role in mushroom growth and quality but can also pose a significant threat to mushroom cultivation. Bacterial diseases can negatively impact *F. filiformis* yield and pose risks to human health (Lomonaco et al., 2009; Scholte et al., 2016; Esposito et al., 2019; U.S. Food and Drug Administration, 2021). Despite the critical role of these microorganisms, the composition, diversity, and pathogenic potential of these bacterial communities within the *F. filiformis* cultivation environment remain insufficiently explored. This study aimed to investigate the bacterial communities associated with *F. filiformis* and its production environment to enhance the understanding of microbial ecology and offer insights for improving agricultural practices, ensuring the safety and quality of mushroom production.

The 16S rRNA metagenomic sequencing revealed a diverse bacterial community within *F. filiformis* cultivation environments, with notable variations between the two industrial-scale facilities studied. The dominant phyla identified were *Proteobacteria* and *Firmicutes*, which included key genera such as *Pseudomonas*, *Lactobacillus*, and *Janthinobacterium*. These genera play diverse roles in mushroom cultivation environments. For example, *Pseudomonas* species are known for their diverse metabolic capabilities, including plant-growth promotion, biocontrol properties, antifungal secondary metabolite production, nutrient cycling, and disease suppression (Palleroni, 2015; Sah et al., 2021; Raaijmakers et al., 2010). However, certain species, such as *P. tolaasii*, are pathogenic and can cause blotch disease in mushrooms (Liu et al., 2022). *Lactobacillus* spp. are recognized for their roles in fermentation processes, potential probiotic benefits, substrate decomposition and pH stabilization through lactic acid production, but their function in mushroom cultivation environments may involve competitive exclusion of pathogens through the production of antimicrobial compounds (Hammes and Hertel, 2015; Kharazian et al., 2017; Murindangabo et al., 2023; Gänzle, 2015; Yang et al., 2018). *Janthinobacterium* spp., known for producing violacein, can exhibit antifungal and antimicrobial properties, which may influence the microbial balance and disease suppression within the cultivation system (Pidot et al., 2014; Pantanella et al., 2007). These results are consistent with previous studies (Benucci and Bonito, 2016; Vos et al., 2017; Carrasco et al., 2019; Shamugam and Kertesz, 2023), which also documented diverse microbial communities in mushroom cultivation settings. Understanding the functional roles of specific taxa within these microbial communities is essential for assessing their impact on mushroom production and human health (Pardo et al., 2013; Chen et al., 2014; Li et al., 2021).

In addition to the pathogenic taxa identified, several beneficial microbial genera were detected, which may play critical roles in promoting the health and productivity of *F. filiformis.* Beneficial bacteria can enhance nutrient availability, stimulate host defenses, and suppress pathogen proliferation through competitive exclusion or antagonistic interactions (Vieira et al., 2023).

Functional profiling through metagenomic or metatranscriptomic analyses could offer deeper insights into the metabolic capabilities of these taxa, helping to identify those that are beneficial or harmful. For example, exploring the roles of *Acinetobacter* and *Flavobacterium* could elucidate their contributions to substrate decomposition and nutrient cycling. *Acinetobacter* species are known for their metabolic versatility, including the degradation of hydrocarbons, while *Flavobacterium* species contribute to the breakdown of polysaccharides and proteins, which may enhance substrate quality and support fungal growth (Jung and Park, 2015; Shrivastava and Berg, 2015; Nemec, 2022). Leveraging these microbes could serve as a natural and sustainable approach to disease management, complementing existing practices and minimizing chemical inputs. The presence of such beneficial communities underscores the importance of maintaining microbial diversity and stability in cultivation environments, as it may contribute to both enhanced yields and reduced disease prevalence.

The rarefaction curve analysis indicated that while most of the bacterial diversity was captured, additional sampling or shotgun metagenomics could provide further insight into rare or low-abundance taxa. The alpha diversity indices (Observed Species, Shannon, Simpson, Chao1, ACE, Goods Coverage, PD Whole Tree) revealed significant differences between microbial communities in Changchun production facility (Group C) and Guizhou production facility (Group G). Higher microbial diversity, particularly in samples C6 and C7 from Changchun production facility, indicated a more stable and diverse microbial ecosystem that may promote healthier mushroom growth and disease suppression. In contrast, Guizhou production facility exhibited lower diversity in samples such as G2 and G5, suggesting potential microbial imbalances and increased susceptibility to pathogens, consistent with findings from previous studies (Carrasco et al., 2019).

The richness, as measured by the Observed Species and Chao1 indices, was higher in Changchun production facility, with samples C6 and C7 demonstrating high evenness and lower dominance of specific species per the Simpson index-indicating a balanced microbial community. Conversely, Guizhou production facility samples like G2 showed lower richness and greater species dominance, suggesting potential vulnerability to pathogens.

The ACE and Simpson indices supported these trends, with consistently higher values in Changchun production facility, reflecting a well-distributed microbial community. Goods Coverage and PD Whole Tree indices indicated a more complete capture of microbial diversity in Changchun production facility, potentially due to better environmental or substrate management. These differences emphasize the role of environmental factors, such as substrate composition, humidity, and management practices, in shaping microbial communities. Higher diversity in Changchun production facility suggests a more resilient microbial ecosystem, critical for effective disease management and enhanced mushroom production.

The beta diversity analysis using the NMDS plot revealed distinct differences in microbial community structures between Changchun production facility and Guizhou production facility. The clear separation of microbial communities suggests that environmental and management practices may significantly influence microbial composition in these *F. filiformis* cultivation environments. Samples from Changchun production facility generally clustered closely, indicating a more consistent and stable microbial community. In contrast, the wider distribution of samples from Guizhou production facility suggests greater variability and potential instability in its microbial communities.

The differences in microbial stability between the two factories may have important implications for disease susceptibility. Group C2 and G2, representing diseased samples, displayed patterns that further highlight these contrasts. Specifically, samples from Group C2 clustered closer to the overall Changchun production facility community structure, suggesting that even diseased samples may retain some community stability. Conversely, Group G2 from Guizhou production facility appeared more separated, consistent with greater microbial variability and potentially indicating higher susceptibility to pathogenic outbreaks. Interestingly, the clustering of healthy samples (C1 and G1) also reflects these patterns. Healthy samples from Changchun production facility (C1) closely clustered with other samples, reinforcing the notion of a stable microbial community that may promote resilience against pathogens. However, healthy samples from Guizhou production facility (G1) exhibited more variability, suggesting a potential imbalance even in ostensibly healthy states.

Overall, the observed differences in microbial community composition and stability highlight the potential influence of environmental factors, such as substrate composition and management practices, on microbial diversity and disease outcomes (Nguyen et al., 2021). The greater variability in Guizhou production facility microbial communities may reflect conditions favoring the emergence of pathogenic genera, emphasizing the need for targeted interventions to promote microbial stability and reduce disease risks in mushroom cultivation (Carrasco and Preston, 2020).

Bacterial disease symptoms can manifest at all stages of mushroom growth, significantly diminishing the marketability of mature mushrooms. The presence of various types of rot, including light brown rot, dark brown rot, black-brown rot, and black rot, on mushroom fruiting bodies underscores the role of microbial communities in disease development. Our findings revealed that diseased samples, such as C2, exhibited fewer taxa at both the phyla and genus levels compared to healthier samples, suggesting a potential loss of beneficial or neutral microorganisms. This reduction in microbial diversity may be driven by the dominance of specific pathogenic taxa, such as *Pseudomonas* and *Lactobacillus*. Such shifts in microbial composition emphasize the need to identify and manage these dominant pathogens to mitigate disease impacts (Han et al., 2012; Wu et al., 2016; Wang et al., 2019, 2022; Yan et al., 2019b; Liu et al., 2020, 2022).

Mushroom discoloration and rot severity are influenced by factors including bacterial population density on mushroom caps, specific bacterial strains, infection timing, and environmental conditions (Cho and Kim, 2003; Soler-Rivas et al., 1999). These variations in symptoms may reflect distinct microbial communities associated with diseased samples, aiding in the identification and rapid diagnosis of potential pathogens. Notably, different bacterial species or even strains of the same species can produce similar or distinct rot symptoms, highlighting the importance of comprehensive documentation. Visual records of these symptoms provide a valuable reference for research and facilitate comparisons with previous cases, contributing to a deeper understanding of disease development and its microbial context (Benucci and Bonito, 2016; Vos et al., 2017; Carrasco et al., 2019).

Our study identified multiple bacterial strains associated with diseased *F. filiformis* samples from Changchun and Bijie. Among these, *E. americana* (B2-1), *P. tolaasii* (PT13), and *J. lividum* (YC2-2) were confirmed as primary pathogens through Koch’s postulates, demonstrating their ability to induce disease symptoms. *E. americana* and *P. tolaasii* are established mushroom pathogens, with the latter known for causing blotch disease (Han et al., 2012; Liu et al., 2018; Osdaghi et al., 2019; Liu et al., 2022; Hamidizade et al., 2023). Notably, *J. lividum* was confirmed as a pathogen, and while its role in mushroom disease is less documented, related species such as *J. agaricidamnosum* are known to cause soft rot in *A. bisporus* (Lincoln et al., 1999; Wein et al., 2023), warranting further investigation into its pathogenic mechanisms and interactions within microbial communities. In contrast, strains like *S. maltophilia* (GZ1-3), *Pseudomonas* sp. (GZ2-3), and *L. amnigena* (GZ2-4) did not exhibit pathogenicity. Their presence in diseased samples suggests potential roles as secondary colonizers or contributors to microbial community shifts under disease conditions, which merit further exploration (Braat et al., 2022).

A comparative analysis of strains from Changchun and Bijie provides insights into potential environmental influences on microbial composition and pathogenicity. The detection of common pathogens, such as *E. americana*, in both locations suggests a broad geographical range and adaptability to different cultivation environments. Identifying primary pathogens like *P. tolaasii* and *E. americana* emphasizes the need for targeted management strategies. These strategies may include monitoring pathogen populations, exploring biological control options, and refining cultivation practices to suppress their spread (Osdaghi et al., 2019).

The absence of *L. monocytogenes* in the sampled factories underscores the effectiveness of implemented food safety practices. This finding particularly significant given the recent multinational outbreak of listeriosis linked to *F. filiformis* imported from South Korea (Pereira et al., 2023). Identifying and addressing potential sources of *Listeria* contamination in *F. filiformis* farms, including factory floor, cultivation materials, substrates, and human contact are crucial (Chen et al., 2014; Li et al., 2021). Strict adherence to hygiene protocols, the implementation of MAP, and the application of HACCP principles are essential to prevent *L. monocytogenes* contamination and ensure product safety and quality. Further research could explore the factors contributing to the absence of *L. monocytogenes* and identify strategies that can be adopted into targeted control measures to minimize potential contamination risk.

The study has several limitations that should be considered. First, the DNA extraction method used may introduce biases in the microbial community analysis. Future studies should explore alternative extraction techniques to minimize these biases and enhance consistency and reliability across different laboratories (Greathouse et al., 2019).

Additionally, focusing on a specific region of the 16S rRNA gene (V4 region) may limit the overall representation of microbial diversity. Exploring additional hypervariable regions of the 16S rRNA gene or adopting a whole genome shotgun metagenomics approach could provide a more comprehensive understanding of the microbial communities associated with *F. filiformis* cultivation.

Furthermore, the study excluded certain production stages, which may affect the interpretation of the results. Including a broader range of production stages, such as harvesting and packaging, would provide a more complete picture of microbial dynamics. The sampling was also conducted during a specific season, potentially limiting the representation of microbial ecology throughout the year. Adopting a Longitudinal sampling could better capture seasonal variations and offer a more comprehensive understanding. Addressing these limitations in future research will contribute to a deeper and more comprehensive understanding of the microbial ecology in *F. filiformis* cultivation, with potential implications for improved mushroom production.

## Conclusion

This study provides valuable insights into the microbial diversity present in *F. filiformis* production facilities, emphasizing the roles of both beneficial and pathogenic bacteria. The findings have significant implications for identifying beneficial microbes that promote rapid growth and high yields in mushroom cultivation, as well as for understanding pathogenic threats. This research serves as a foundation for developing guidelines to implement Mushroom Good Agricultural Practices (MGAP) and designing critical control points in *F. filiformis* facility operations.

Metagenomics surveillance has proven to be a powerful tool for comprehensively monitoring microbial communities, enabling early detection of potential pathogens and facilitating the implementation of targeted control measures. Incorporating metagenomics surveillance into mushroom production practices can optimize cultivation techniques, improve disease management, and enhance food safety in *F. filiformis* operations. Overall, this research reveals important microbial dynamics in *F. filiformis* farms and offers practical insights for optimizing cultivation, disease management, and food safety, contributing to the sustainability and efficiency of mushroom production.

## Data Availability

The datasets presented in this study can be found in online repositories. The names of the repository/repositories and accession number(s) can be found in the article/Supplementary material.

## Footnotes

^1^ http://cutadapt.readthedocs.io/en/stable/

^2^ https://github.com/torognes/vsearch/

^3^ http://www.drive5.com/uparse/

^4^ http://www.arb-silva.de/

^5^ https://blast.ncbi.nlm.nih.gov/Blast.cgi

## References

[ref1] Benucci G. M. N. Bonito G. M. (2016). The truffle microbiome: species and geography effects on bacteria associated with fruiting bodies of hypogeous pezizales. Microb. Ecol. 72, 4–8. doi: 10.1007/s00248-016-0755-3, PMID: 27026101

[ref2] Berendsen R. L. Kalkhove S. I. C. Lugones L. G. Wösten H. A. B. Bakker P. A. H. M. (2012). Germination of *Lecanicillium fungicola* in the mycosphere of *Agaricus bisporus*: spore germination of *Lecanicillium fungicola*. Environ. Microbiol. Rep. 4, 227–233. doi: 10.1111/j.1758-2229.2011.00325.x, PMID: 23757277

[ref3] Braat N. Koster M. C. Wösten H. A. B. (2022). Beneficial interactions between bacteria and edible mushrooms. Fungal Biol. Rev. 39, 60–72. doi: 10.1016/j.fbr.2021.12.001

[ref4] Caporaso J. G. Lauber C. L. Walters W. A. Berg-Lyons D. Lozupone C. A. Turnbaugh P. J.. (2011). Global patterns of 16S rRNA diversity at a depth of millions of sequences per sample. Proc. Natl. Acad. Sci. USA 108, 4516–4522. doi: 10.1073/pnas.1000080107, PMID: 20534432 PMC3063599

[ref5] Carrasco J. Preston G. M. (2020). Growing edible mushrooms: a conversation between bacteria and fungi. Environ. Microbiol. 22, 858–872. doi: 10.1111/1462-2920.14765, PMID: 31361932

[ref6] Carrasco J. Tello M. L. de Toro M. Tkacz A. Poole P. Preston G. (2019). Casing microbiome dynamics during button mushroom cultivation: implications for dry and wet bubble diseases. Microbiology 165, 611–624. doi: 10.1099/mic.0.000792, PMID: 30994437

[ref7] Chen M. Wu Q. Zhang J. Guo W. Wu S. Yang X. (2014). Prevalence and contamination patterns of *Listeria monocytogenes* in *Flammulina velutipes* plants. Foodborne Pathog. Dis. 11, 620–627. doi: 10.1089/fpd.2013.1727 24824447

[ref8] Cho K. H. Kim Y. K. (2003). Two types of ion channel formation of tolaasin, a Pseudomonas peptide toxin. FEMS Microbiol. Lett. 221, 221–226. doi: 10.1016/S0378-1097(03)00182-4 12725930

[ref9] Clarke J. D. (2009). Cetyltrimethyl ammonium bromide (CTAB) DNA miniprep for plant DNA isolation. Cold Spring Harb. Protoc. 2009:pdb.prot5177. doi: 10.1101/pdb.prot5177 20147112

[ref10] Dong Y. Miao R. Feng R. Wang T. Yan J. Zhao X.. (2022). Edible and medicinal fungi breeding techniques, a review: current status and future prospects. Curr. Res. Food Sci. 5, 2070–2080. doi: 10.1016/j.crfs.2022.09.002, PMID: 36387595 PMC9640942

[ref11] Edgar R. C. (2013). UPARSE: highly accurate OTU sequences from microbial amplicon reads. Nat. Methods 10, 996–998. doi: 10.1038/nmeth.2604, PMID: 23955772

[ref12] Esposito S. Miconi F. Molinari D. Savarese E. Celi F. Marchese L.. (2019). What is the role of *Ewingella americana* in humans? A case report in a healthy 4-year-old girl. BMC Infect. Dis. 19:386. doi: 10.1186/s12879-019-4021-4, PMID: 31060497 PMC6501419

[ref13] Gänzle M. G. (2015). Lactic metabolism revisited: metabolism of lactic acid bacteria in food fermentations and food spoilage. Curr. Opin. Food Sci. 2, 106–117. doi: 10.1016/j.cofs.2015.03.001

[ref14] Greathouse K. L. Sinha R. Vogtmann E. (2019). DNA extraction for human microbiome studies: the issue of standardization. Genome Biol. 20:212. doi: 10.1186/s13059-019-1843-8, PMID: 31639026 PMC6802309

[ref15] Haas B. J. Gevers D. Earl A. M. Feldgarden M. Ward D. V. Giannoukos G.. (2011). Chimeric 16S rRNA sequence formation and detection in sanger and 454-pyrosequenced PCR amplicons. Genome Res. 21, 494–504. doi: 10.1101/gr.112730.110, PMID: 21212162 PMC3044863

[ref16] Hall T. (1999). Bioedit: A user-Friendly biological sequence alignment editor and analysis program for windows 95/98/NT. doi: 10.14601/Phytopathol_Mediterr-14998u1.29

[ref17] Hamidizade M. Taghavi S. M. Moallem M. Aeini M. Fazliarab A. Abachi H.. (2023). *Ewingella americana*: an emerging multifaceted pathogen of edible mushrooms. Phytopathology 113, 150–159. doi: 10.1094/PHYTO-08-22-0299-R, PMID: 36131391

[ref18] Hammes W. P. Hertel C. (2015). “Lactobacillus†” in Bergey's manual of systematics of Archaea and Bacteria. eds. Trujillo M. E. Dedysh S. DeVos P. Hedlund B. Kämpfer P. Rainey F. A.

[ref19] Han H. S. Jhune C. S. Cheong J. C. Oh J. A. Kong W. S. Cha J. S.. (2012). Occurrence of black rot of cultivated mushrooms (*Flammulina velutipes*) caused by *Pseudomonas tolaasii* in Korea. Eur. J. Plant Pathol. 133, 527–535. doi: 10.1007/s10658-012-9941-4

[ref20] He Y. Caporaso J. G. Jiang X. T. Sheng H. F. Huse S. M. Rideout J. R.. (2015). Stability of operational taxonomic units: an important but neglected property for analyzing microbial diversity. Microbiome 3:20. doi: 10.1186/s40168-015-0081-x, PMID: 25995836 PMC4438525

[ref21] Hess M. Sczyrba A. Egan R. Kim T. W. Chokhawala H. Schroth G.. (2011). Metagenomic discovery of biomass-degrading genes and genomes from cow rumen. Science 331, 463–467. doi: 10.1126/science.1200387, PMID: 21273488

[ref22] Jayaraman S. Yadav B. Dalal R. C. Naorem A. Sinha N. K. Rao C. S.. (2024). Mushroom farming: a review focusing on soil health, nutritional security and environmental sustainability. Farm. Syst. 2:100098. doi: 10.1016/j.farsys.2024.100098

[ref23] Jin S. Zhou J. Ye J. (2008). Adoption of HACCP system in the Chinese food industry: a comparative analysis. Food Control 19, 823–828. doi: 10.1016/j.foodcont.2008.01.008

[ref24] Jung J. Park W. (2015). Acinetobacter species as model microorganisms in environmental microbiology: current state and perspectives. Appl. Microbiol. Biotechnol. 99, 2533–2548. doi: 10.1007/s00253-015-6439-y, PMID: 25693672

[ref25] Kalač P. (2013). A review of chemical composition and nutritional value of wild-growing and cultivated mushrooms: chemical composition of edible mushrooms. J. Sci. Food Agric. 93, 209–218. doi: 10.1002/jsfa.5960, PMID: 23172575

[ref26] Kharazian Z. A. Jouzani G. S. Aghdasi M. Khorvash M. Zamani M. Mohammadzadeh H. (2017). Biocontrol potential of Lactobacillus strains isolated from corn silages against some plant pathogenic fungi. Biol. Control 110, 33–43. doi: 10.1016/j.biocontrol.2017.04.004

[ref27] Kolisko M. Flegontova O. Karnkowska A. Lax G. Maritz J. M. Pánek T.. (2020). EukRef-excavates: seven curated SSU ribosomal RNA gene databases. Database (Oxford) 2020:baaa080. doi: 10.1093/database/baaa080, PMID: 33216898 PMC7678783

[ref28] Lane D. J. (1991). “16S/23S rRNA sequencing” in Nucleic Acid Techniques in Bacterial Systematics (New York: John Wiley and Sons), 115–175.

[ref29] Li C. Xu S. (2022). Edible mushroom industry in China: current state and perspectives. Appl. Microbiol. Biotechnol. 106, 3949–3955. doi: 10.1007/s00253-022-11985-0, PMID: 35622125

[ref30] Li F. Ye Q. Chen M. Zhang J. Xue L. Wang J.. (2021). Multiplex PCR for the identification of pathogenic Listeria in *Flammulina velutipes* plant based on novel specific targets revealed by Pan-genome analysis. Front. Microbiol. 11:634255. doi: 10.3389/fmicb.2020.634255, PMID: 33519795 PMC7843925

[ref31] Lincoln S. P. Fermor T. R. Tindall B. J. (1999). *Janthinobacterium agaricidamnosum* sp. nov., a soft rot pathogen of *Agaricus bisporus*. Int. J. Syst. Bacteriol. 49, 1577–1589. doi: 10.1099/00207713-49-4-1577, PMID: 10555339

[ref32] Liu Z. Sheng H. Okorley B. A. Li Y. Sossah F. L. (2020). Comparative genomic analysis provides insights into the phylogeny, Resistome, Virulome, and host adaptation in the genus Ewingella. Pathogens 9:330. doi: 10.3390/pathogens9050330, PMID: 32354059 PMC7281767

[ref33] Liu Z. H. Sossah F. L. Li Y. Fu Y. P. (2018). First report of *Ewingella americana* causing bacterial Brown rot disease on cultivated needle mushroom (*Flammulina velutipes*) in China. Plant Dis. 102:2633. doi: 10.1094/PDIS-02-18-0351-PDN

[ref34] Liu Z. Zhao Y. Sossah F. L. Okorley B. A. Amoako D. G. Liu P.. (2022). Characterization, pathogenicity, phylogeny, and comparative genomic analysis of *Pseudomonas tolaasii* strains isolated from various mushrooms in China. Phytopathology 112, 521–534. doi: 10.1094/PHYTO-12-20-0550-R 34293910

[ref35] Liu Z. L. Zhou S. Zhang W. Wu S. Chen X. Wang X.. (2020). First report of *Cedecea neteri* causing yellow rot disease in *Pleurotus pulmonarius* in China. Plant Dis. 105:1189. doi: 10.1094/PDIS-09-20-1886-PDN

[ref36] Lomonaco S. Decastelli L. Nucera D. Gallina S. Manila Bianchi D. Civera T. (2009). *Listeria monocytogenes* in Gorgonzola: subtypes, diversity and persistence over time. Int. J. Food Microbiol. 128, 516–520. doi: 10.1016/j.ijfoodmicro.2008.10.009, PMID: 18990461

[ref37] Martin M. (2011). Cutadapt removes adapter sequences from high-throughput sequencing reads. EMBnet J. 17, 10–12. doi: 10.14806/EJ.17.1.200

[ref38] Mergaert J. Verdonck L. Kersters K. (1993). Transfer of Erwinia ananas (synonym, *Erwinia uredovora*) and *Erwinia stewartii* to the genus Pantoea emend. As Pantoea ananas (serrano 1928) comb. nov. and *Pantoea stewartii* (smith 1898) comb, nov., respectively, and description of *Pantoea stewartii* subsp. indologenes subsp. nov. Int. J. Syst. Bacteriol. 43, 162–173. doi: 10.1099/00207713-43-1-162

[ref39] Murindangabo Y. T. Kopecký M. Perná K. Nguyen T. G. Konvalina P. Kavková M. (2023). Prominent use of lactic acid bacteria in soil-plant systems. Appl. Soil Ecol. 189:104955. doi: 10.1016/j.apsoil.2023.104955

[ref40] Nemec A. (2022). “Acinetobacter†.” in Bergey's manual of systematics of Archaea and Bacteria. eds. Trujillo M. E. Dedysh S. DeVos P. Hedlund B. Kämpfer P. Rainey F. A.

[ref41] Nguyen J. Lara-Gutiérrez J. Stocker R. (2021). Environmental fluctuations and their effects on microbial communities, populations and individuals. FEMS Microbiol. Rev. 45:fuaa068. doi: 10.1093/femsre/fuaa068, PMID: 33338228 PMC8371271

[ref42] Oksanen J. Blanchet F. G. Friendly M. Kindt R. Wagner H. H. (2020). Vegan community ecology package version 2.5–7 November 2020.

[ref43] Osdaghi E. Martins S. J. Ramos-Sepulveda L. Vieira F. R. Pecchia J. A. Beyer D. M.. (2019). 100 years since Tolaas: bacterial blotch of mushrooms in the 21^st^ century. Plant Dis. 103, 2714–2732. doi: 10.1094/PDIS-03-19-0589-FE, PMID: 31560599

[ref44] Palleroni N. J. (2015). “Pseudomonas^†^” in Bergey's manual of systematics of Archaea and Bacteria. eds. Trujillo M. E. Dedysh S. DeVos P. Hedlund B. Kämpfer P. Rainey F. A.

[ref45] Pantanella F. Berlutti F. Passariello C. Sarli S. Morea C. Schippa S. (2007). Violacein and biofilm production in *Janthinobacterium lividum*. J. Appl. Microbiol. 102, 992–999. doi: 10.1111/j.1365-2672.2006.03155.x 17381742

[ref46] Pardo J. E. De Figueirêdo V. R. Álvarez-Ortí M. Zied D. C. Peñaranda J. A. Dias E. S.. (2013). Application of Hazard analysis and critical control points (HACCP) to the cultivation line of mushroom and other cultivated edible Fungi. Indian J. Microbiol. 53, 359–369. doi: 10.1007/s12088-013-0365-4, PMID: 24426137 PMC3689402

[ref47] Pereira E. Conrad A. Tesfai A. Palacios A. Kandar R. Kearney A.. (2023). Multinational outbreak of *Listeria monocytogenes* infections linked to Enoki mushrooms imported from the Republic of Korea 2016–2020. J. Food Protect. 86:100101. doi: 10.1016/j.jfp.2023.100101, PMID: 37169291 PMC10947956

[ref48] Pidot S. J. Coyne S. Kloss F. Hertweck C. (2014). Antibiotics from neglected bacterial sources. Int. J. Med. Microbiol. 304, 14–22. doi: 10.1016/j.ijmm.2013.08.011 24120363

[ref49] Quast C. Pruesse E. Yilmaz P. Gerken J. Schweer T. Yarza P.. (2013). The SILVA ribosomal RNA gene database project: improved data processing and web-based tools. Nucleic Acids Res. 41, D590–D596. doi: 10.1093/nar/gks1219, PMID: 23193283 PMC3531112

[ref50] Raaijmakers J. M. De Bruijn I. Nybroe O. Ongena M. (2010). Natural functions of lipopeptides from Bacillus and Pseudomonas: more than surfactants and antibiotics. FEMS Microbiol. Rev. 34, 1037–1062. doi: 10.1111/j.1574-6976.2010.00221.x, PMID: 20412310

[ref51] Rahman M. A. Abdullah N. Aminudin N. (2015). Antioxidative effects and inhibition of human Low density lipoprotein oxidation *in vitro* of polyphenolic compounds in *Flammulina velutipes* (Golden needle mushroom). Oxidative Med. Cell. Longev. 2015, 1–10. doi: 10.1155/2015/403023, PMID: 26180589 PMC4477244

[ref52] Rognes T. Flouri T. Nichols B. Quince C. Mahé F. (2016). VSEARCH: a versatile open source tool for metagenomics. PeerJ 4:e2584. doi: 10.7717/peerj.2584, PMID: 27781170 PMC5075697

[ref53] Royse D. J. Baars J. J. P. Tan Q. (2017). “Current overview of mushroom production in the world” in Edible and Medicinal Mushrooms: Technology and Applications, vol. 2.

[ref54] Sah S. Krishnani S. Singh R. (2021). Pseudomonas mediated nutritional and growth promotional activities for sustainable food security. Curr. Res. Microb. Sci. 2:100084. doi: 10.1016/j.crmicr.2021.100084, PMID: 34917993 PMC8645841

[ref55] Sangeeta Sharma D. Ramniwas S. Mugabi R. Uddin J. Nayik G. A. (2024). Revolutionizing mushroom processing: innovative techniques and technologies. Food Chem. X 23:101774. doi: 10.1016/j.fochx.2024.101774, PMID: 39280230 PMC11402429

[ref56] Schloss P. D. Westcott S. L. Ryabin T. Hall J. R. Hartmann M. Hollister E. B.. (2009). Introducing mothur: open-source, platform-independent, community-supported software for describing and comparing microbial communities. Appl. Environ. Microbiol. 75, 7537–7541. doi: 10.1128/AEM.01541-09, PMID: 19801464 PMC2786419

[ref57] Scholte J. B. J. Zhou T. L. Bergmans D. C. J. J. Rohde G. G. U. Winkens B. Van Dessel H. A.. (2016). *Stenotrophomonas maltophilia* ventilator-associated pneumonia. A retrospective matched case-control study. Infect. Dis. (Lond.) 48, 738–743. doi: 10.1080/23744235.2016.1185534 27207483

[ref58] Shamugam S. Kertesz M. A. (2023). Bacterial interactions with the mycelium of the cultivated edible mushrooms *Agaricus bisporus* and *Pleurotus ostreatus*. J. Appl. Microbiol. 134:lxac018. doi: 10.1093/jambio/lxac018, PMID: 36626759

[ref59] Shrivastava A. Berg H. C. (2015). Towards a model for Flavobacterium gliding. Curr. Opin. Microbiol. 28, 93–97. doi: 10.1016/j.mib.2015.07.018, PMID: 26476806 PMC4688146

[ref60] Soler-Rivas C. Jolivet S. Arpin N. Olivier J. M. Wichers H. J. (1999). Biochemical and physiological aspects of brown blotch disease of *Agaricus bisporus*. FEMS Microbiol. Rev. 23, 591–614. doi: 10.1111/j.1574-6976.1999.tb00415.x 10525168

[ref61] Su J. Ji W. Sun X. Wang H. Kang Y. Yao B. (2023). Effects of different management practices on soil microbial community structure and function in alpine grassland. J. Environ. Manag. 327:116859. doi: 10.1016/j.jenvman.2022.116859, PMID: 36450164

[ref62] Tamura K. Nei M. Kumar S. (2004). Prospects for inferring very large phylogenies by using the neighbor-joining method. Proc. Natl. Acad. Sci. USA 101, 11030–11035. doi: 10.1073/pnas.0404206101, PMID: 15258291 PMC491989

[ref63] Tamura K. Stecher G. Kumar S. (2021). MEGA11: molecular evolutionary genetics analysis version 11. Mol. Biol. Evol. 38, 3022–3027. doi: 10.1093/molbev/msab120, PMID: 33892491 PMC8233496

[ref64] Tang C. Hoo P. C. X. Tan L. T. H. Pusparajah P. Khan T. M. Lee L. H.. (2016). Golden needle mushroom: a culinary medicine with evidenced-based biological activities and health promoting properties. Front. Pharmacol. 7:474. doi: 10.3389/fphar.2016.00474 28003804 PMC5141589

[ref65] Thompson J. D. Higgins D. G. Gibson T. J. (1994). CLUSTAL W: improving the sensitivity of progressive multiple sequence alignment through sequence weighting, position-specific gap penalties and weight matrix choice. Nucleic Acids Res. 22, 4673–4680. doi: 10.1093/nar/22.22.4673, PMID: 7984417 PMC308517

[ref66] U.S. Food and Drug Administration. (2021). Outbreak investigation of Listeria monocytogenes: Dole packaged salad (December 2021). FDA. Available at: https://www.fda.gov/food/outbreaks-foodborne-illness/outbreak-investigation-listeria-monocytogenes-dole-packaged-salad-december-2021 (Accessed July 29, 2022).

[ref67] Vieira F. R. Di Tomassi I. O'Connor E. Bull C. T. Pecchia J. A. Hockett K. L. (2023). Manipulating *Agaricus bisporus* developmental patterns by passaging microbial communities in complex substrates. Microbiol. Spectr. 11:e0197823. doi: 10.1128/spectrum.01978-23, PMID: 37831469 PMC10714785

[ref68] Vos A. M. Heijboer A. Boschker H. T. S. Bonnet B. Lugones L. G. Wösten H. A. B. (2017). Microbial biomass in compost during colonization of *Agaricus bisporus*. AMB Express 7:12. doi: 10.1186/s13568-016-0304-y, PMID: 28050852 PMC5209305

[ref69] Wang Q. Garrity G. M. Tiedje J. M. Cole J. R. (2007). Naïve Bayesian classifier for rapid assignment of rRNA sequences into the new bacterial taxonomy. Appl. Environ. Microbiol. 73, 5261–5267. doi: 10.1128/AEM.00062-07, PMID: 17586664 PMC1950982

[ref70] Wang Q. Guo M. Xu R. Zhang J. Bian Y. Xiao Y. (2019). Transcriptional changes on blight fruiting body of Flammulina velutipes caused by two new bacterial pathogens. Front. Microbiol. 10:2845. doi: 10.3389/fmicb.2019.02845, PMID: 31921028 PMC6917577

[ref71] Wang P. M. Liu X. B. Dai Y. C. Horak E. Steffen K. Yang Z. L. (2018). Phylogeny and species delimitation of Flammulina: taxonomic status of winter mushroom in East Asia and a new European species identified using an integrated approach. Mycol. Prog. 17, 1013–1030. doi: 10.1007/s11557-018-1409-2

[ref72] Wang Q. Xu R. Guo M. Shen N. Chuaoen P. Qiu K.. (2022). Serial transcriptional changes of *Flammulina filiformis* (winter mushroom) mycelia infected by *Pseudomonas migulae*. Sci. Hortic. 297:110965. doi: 10.1016/j.scienta.2022.110965

[ref73] Wei Q. Pan X. Li J. Jia Z. Fang T. Jiang Y. (2021). Isolation and molecular identification of the native microflora on *Flammulina velutipes* fruiting bodies and modeling the growth of dominant microbiota (*Lactococcus lactis*). Front. Microbiol. 12:664874. doi: 10.3389/fmicb.2021.664874 34093480 PMC8176924

[ref74] Wein P. Dornblut K. Herkersdorf S. Krüger T. Molloy E. M. Brakhage A. A.. (2023). Bacterial secretion systems contribute to rapid tissue decay in button mushroom soft rot disease. MBio 14:e0078723. doi: 10.1128/mbio.00787-23, PMID: 37486262 PMC10470514

[ref75] Wu Z. Peng W. He X. Wang B. Gan B. Zhang X. (2016). Mushroom tumor: a new disease on *Flammulina velutipes* caused by *Ochrobactrum pseudogrignonense*. FEMS Microbiol. Lett. 363:fnv226. doi: 10.1093/femsle/fnv226, PMID: 26667221

[ref77] Yan J. J. Tong Z. J. Liu Y. Y. Li Y. N. Zhao C. Mukhtar I.. (2019a). Comparative transcriptomics of Flammulina filiformis suggests a high CO_2_ concentration inhibits early pileus expansion by decreasing cell division control pathways. Int. J. Mol. Sci. 20:5923. doi: 10.3390/ijms20235923, PMID: 31775357 PMC6929049

[ref76] Yan J. J. Lin Z. Y. Wang R. Q. Liu F. Tong Z. J. Jiang Y. J.. (2019b). First report of *Erwinia persicina* causing pink disease in *Flammulina velutipes* (Enoki mushroom) in China. Plant Dis. 103:1014. doi: 10.1094/PDIS-06-18-0950-PDN

[ref78] Yang K. Xu M. Zhong F. Zhu J. (2018). Rapid differentiation of Lactobacillus species via metabolic profiling. J. Microbiol. Methods 154, 147–155. doi: 10.1016/j.mimet.2018.10.013, PMID: 30359661

[ref79] Youssef N. Sheik C. S. Krumholz L. R. Najar F. Z. Roe B. A. Elshahed M. S. (2009). Comparison of species richness estimates obtained using nearly complete fragments and simulated pyrosequencing-generated fragments in 16S rRNA gene-based environmental surveys. Appl. Environ. Microbiol. 75, 5227–5236. doi: 10.1128/AEM.00592-09, PMID: 19561178 PMC2725448

